# Supplementary material for: Elucidation of transient protein-protein interactions within carrier protein-dependent biosynthesis
Source: Commun Biol. 2021 Mar 16;4:340. doi: 10.1038/s42003-021-01838-3 (PMC7966745; doi:10.1038/s42003-021-01838-3)
Supplement: Supplementary file 2 — Supplementary Information [file 42003_2021_1838_MOESM2_ESM.pdf]

Supplementary Materials for

Elucidation of transient protein-protein interactions within carrier protein-dependent biosynthesis

Thomas G. Bartholow[1], Terra Sztain[1], Ashay Patel[1], D. John Lee[1,3], Megan A. Young[1], Ruben Abagyan[2], Michael D. Burkart[1*]

*Corresponding author. Email: mburkart@ucsd.edu

This PDF file includes:

Supplementary Figures

S1 ^{1}H-^{15}N HSQC titration of C8-AcpP with FabI

S2 ^{1}H-^{15}N HSQC titration of C8-AcpP with FabG

S3 ^{1}H-^{15}N HSQC titration of C8-AcpP with TesA

S4 Electrostatic binding surface of each partner enzyme

S5 Interfaces of each partner studied

S6 ^{1}H-^{15}N HSQC assignment of C8-AcpP

S7 Purification of FabG

S8 Purification of FabI

S9 Purification of FabF

S10 Purification of TesA

S11 Purification of AcpP

S12 Titan peak fitting of FabF titration data

S13 Titan peak fitting of FabG titration data

S14 Titan peak fitting of FabI titration data

S15 Titan peak fitting of TesA titration data

Supplementary Tables

S1 Interface and global alignment scores for docked models

S2 Table of the docked partner characteristics.

S3 Peak list of zero point and saturated FabF titration points

S4 Peak list of zero point and saturated FabI titration points

S5 Peak list of zero point and saturated FabG titration points

S6 Peak list of zero point and saturated TesA titration points

S7 Comparisons of the residue informed and uninformed top 10 docking poses.

S8 Comparisons of online docking resources' ability to recreate the FabF-AcpP crosslinked interface

S9 Thermodynamic and Kinetic parameters of the elongating enzymes and a thioesterase of E. coli Fatty Acid Biosynthesis binding an amide linked octanoyl AcpP

Supplementary Figures

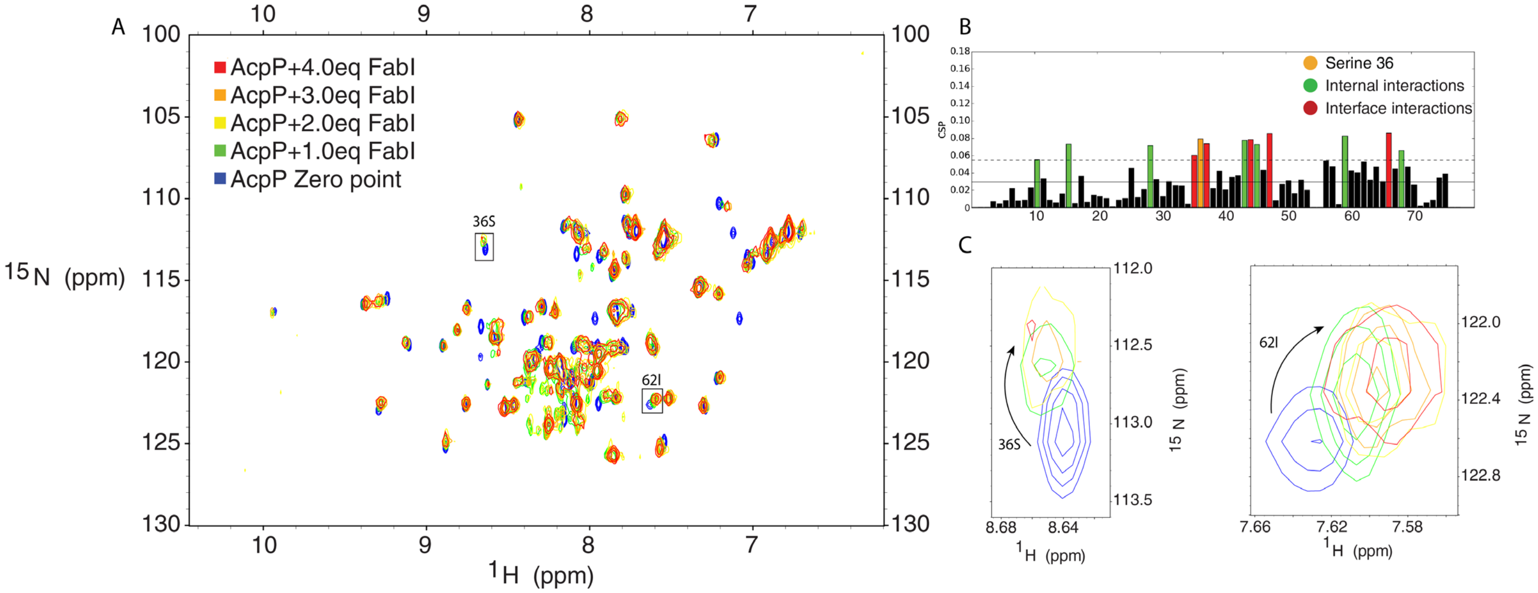

Figure S1. ^{1}H-^{15}N HSQC titration of C8-AcpP with FabI A) 5 overlayed ^{1}H-^{15}N HSQC spectra of ^{15}N-C8-AcpP titrated with increasing concentrations of unlabeled FabI. B) Bar chart of each AcpP residue's CSP with 4.0 molar equivalents of FabI. The mean is shown as a solid line and one standard deviation is the dashed line above. CSPs greater than this value are shown in colors as described by the insert. C) Two examples of residues displaying "titration curves," zoomed in from the boxed residues on the full spectra.

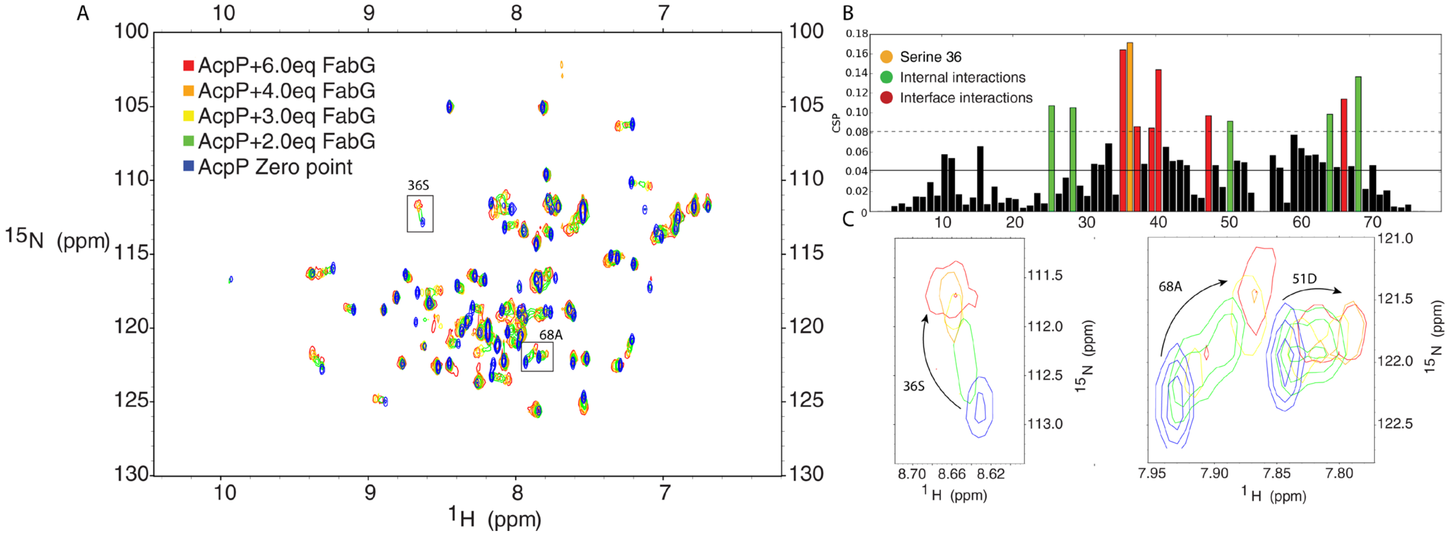

Figure S2 ^{1}H-^{15}N HSQC titration of C8-AcpP with FabG A) 5 overlayed ^{1}H-^{15}N HSQC spectra of ^{15}N-C8-AcpP titrated with increasing concentrations of unlabeled FabG. B) Bar chart of each AcpP residue’s CSP with 6.0 molar equivalents of FabG, The mean is shown as a solid line and one standard deviation is the dashed line above. CSPs greater than this value are shown in colors as described by the insert. C) Two examples of residues displaying “titration curves,” zoomed in from the boxed residues on the full spectra.

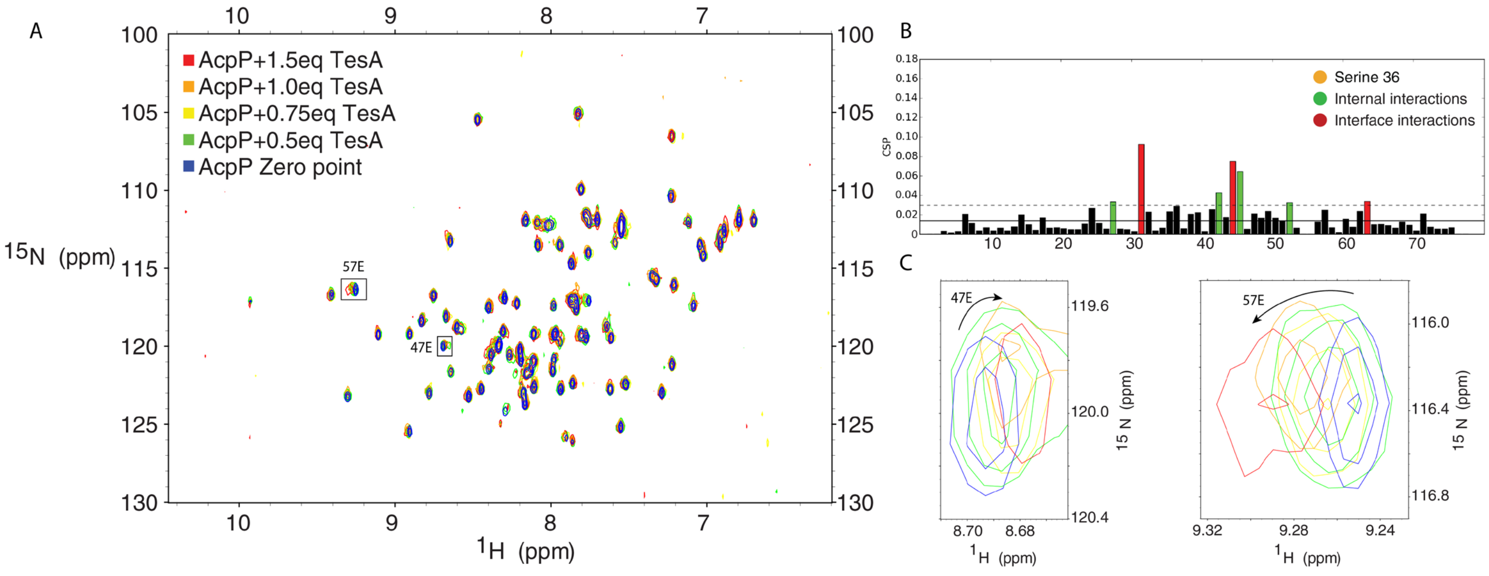

Figure S3 ^{1}H-^{15}N HSQC titration of C8-AcpP with TesA A) 5 overlayed ^{1}H-^{15}N HSQC spectra of ^{15}N-C8-AcpP titrated with increasing concentrations of unlabeled TesA. B) Bar chart of each AcpP residue's CSP with 1.5 molar equivalents of TesA, The mean is shown as a solid line and one standard deviation is the dashed line above. CSPs greater than this value are shown in colors as described by the insert. C) Two examples of residues displaying "titration curves," zoomed in from the boxed residues on the full spectra.

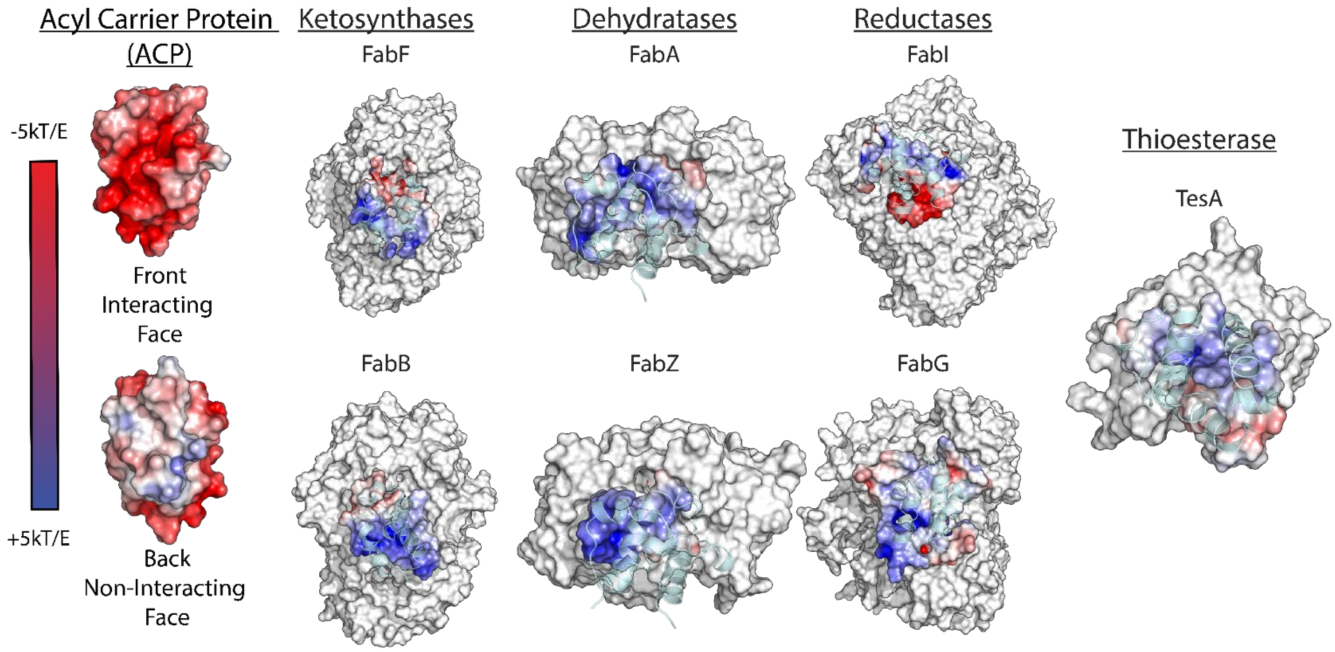

Figure S4 Electrostatic binding surface of each partner enzyme. The surfaces of each of the 7 partner proteins from 4 families of enzymes and the AcpP's interacting and non-interacting face. APBS electrostatics are shown for residues within 5 Å of AcpP in the docked model. The entire AcpP is displayed with the calculated electrostatics.

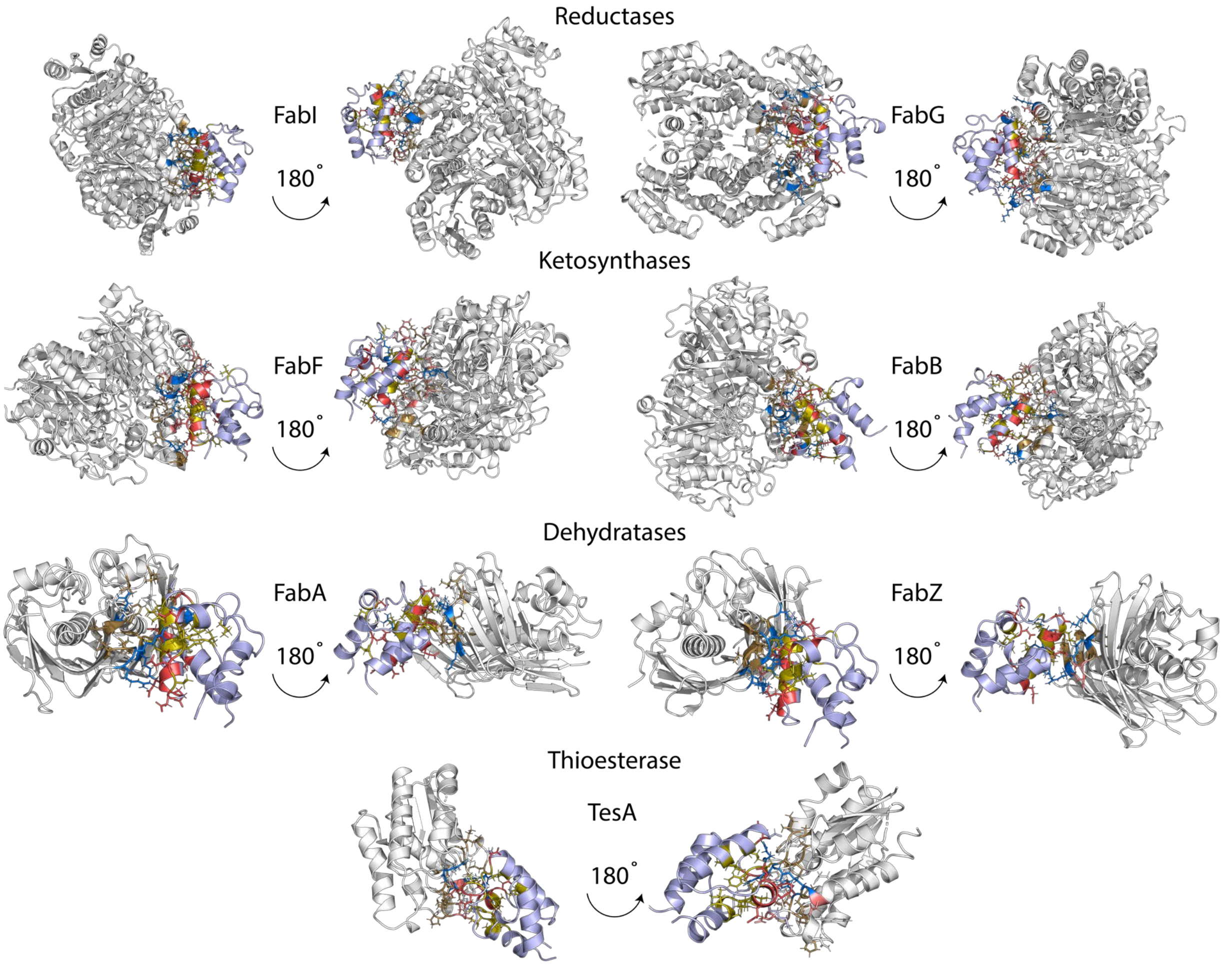

Figure S5 Interfaces of each partner studied. Two orientations of the interface of each enzyme studied in this work. Partner proteins are showed in gray with the AcpP in light purple. Negatively charged residues at the interface are colored red with positively charged residues showed in blue. Hydrophobic residues on the AcpP which lie at the interface are colored yellow while hydrophobic residues on the partner protein are tan.

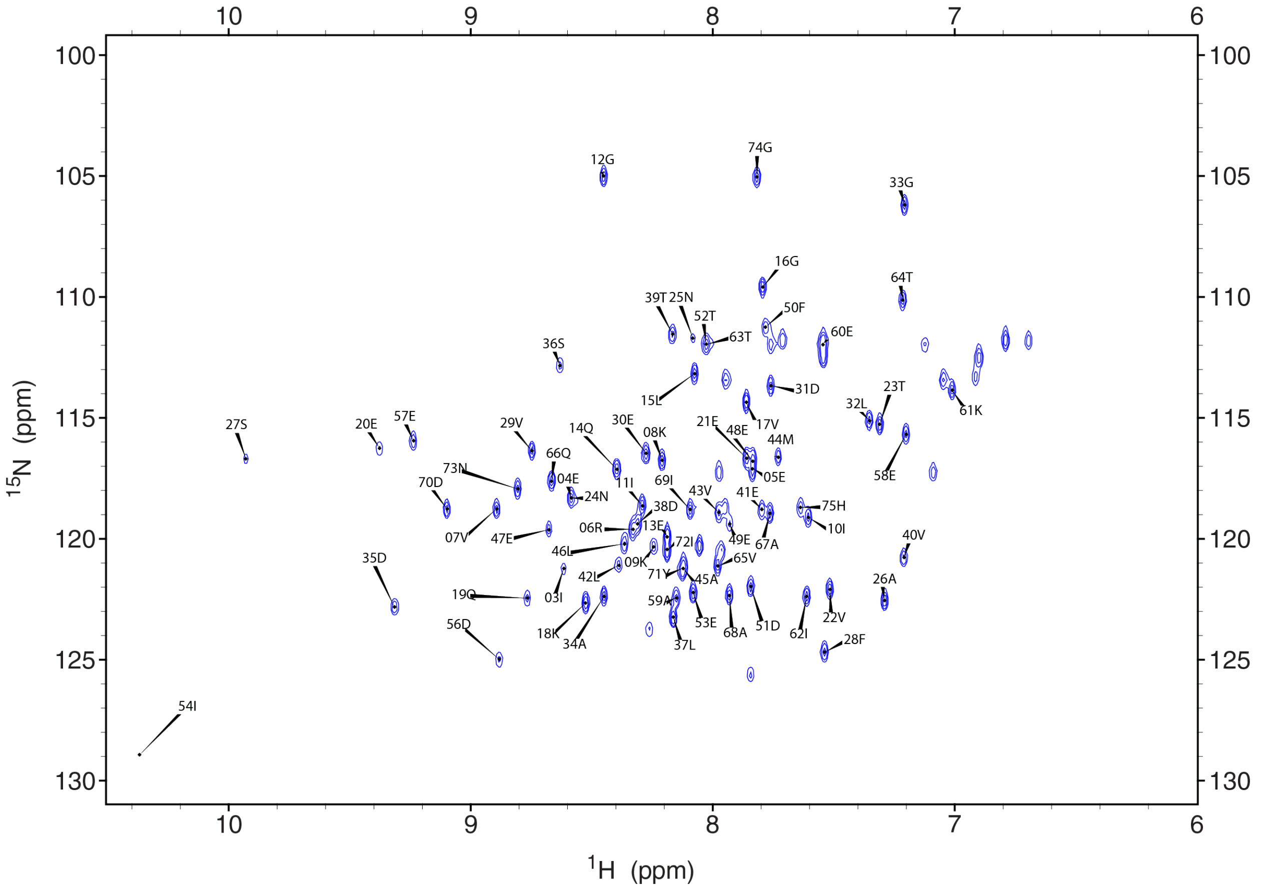

Figure S6 Residue assignments of C8-AcpP. Assignments were taken from a previous study performed in the Burkart lab[31] and used to assign the four titrations performed.

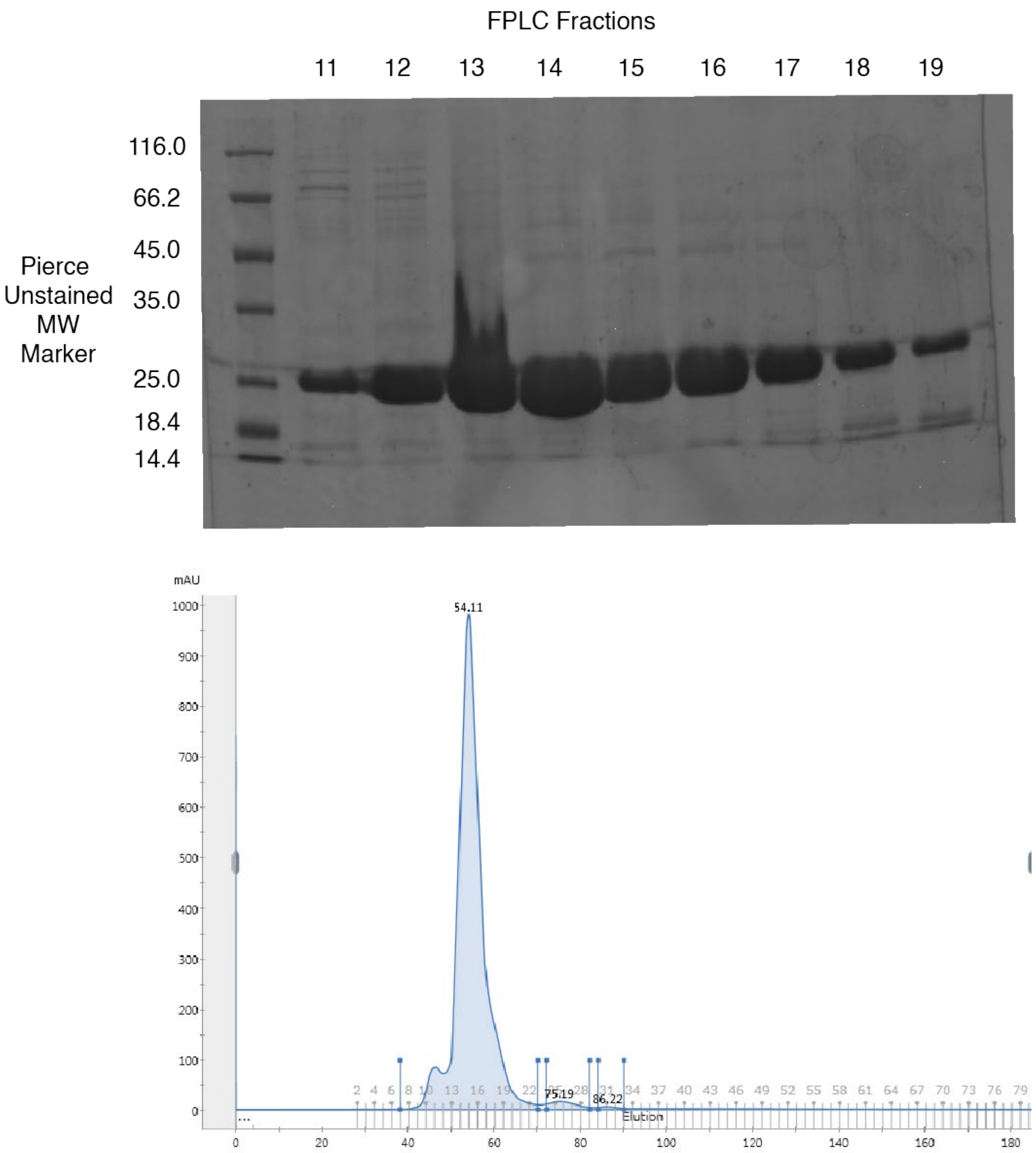

Figure S7 Purification of FabG. FabG was purified as described in the methods. A 12% acrylamide denaturing SDS gel was run on the primary peak identified by the FPLC to verify the identity of the FabG.

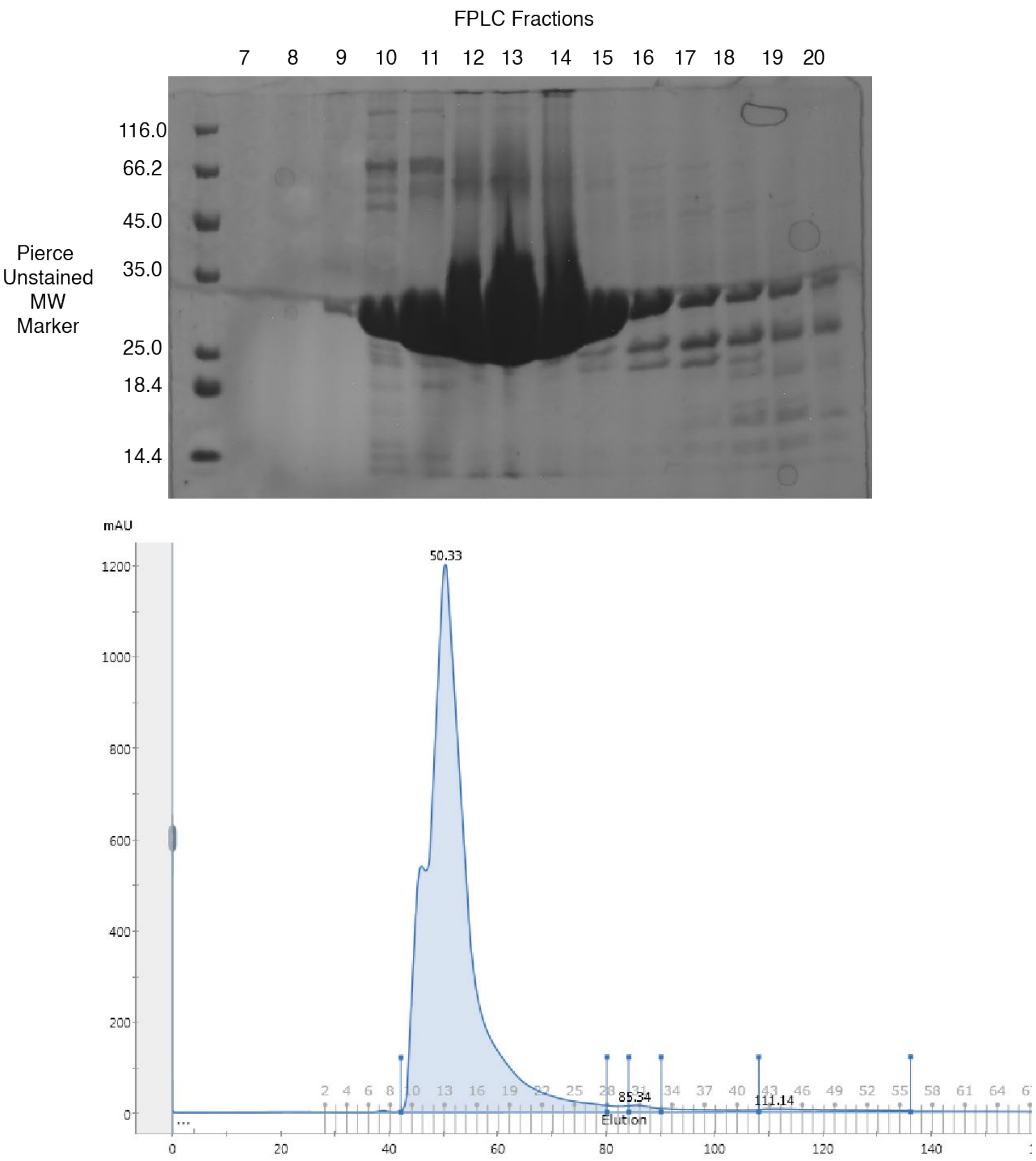

Figure S8 Purification of FabI. FabI was purified by the standard method described in the methods. After FPLCing samples were chosen to run on an SDS gel to verify the purity of the protein. A 12% acrylamide denaturing SDS gel was run, showing very large quantities of protein purified for the experiment. Fractions 12, 13, and 14 were taken as the center of the peak and due to the high concentration of protein already present.

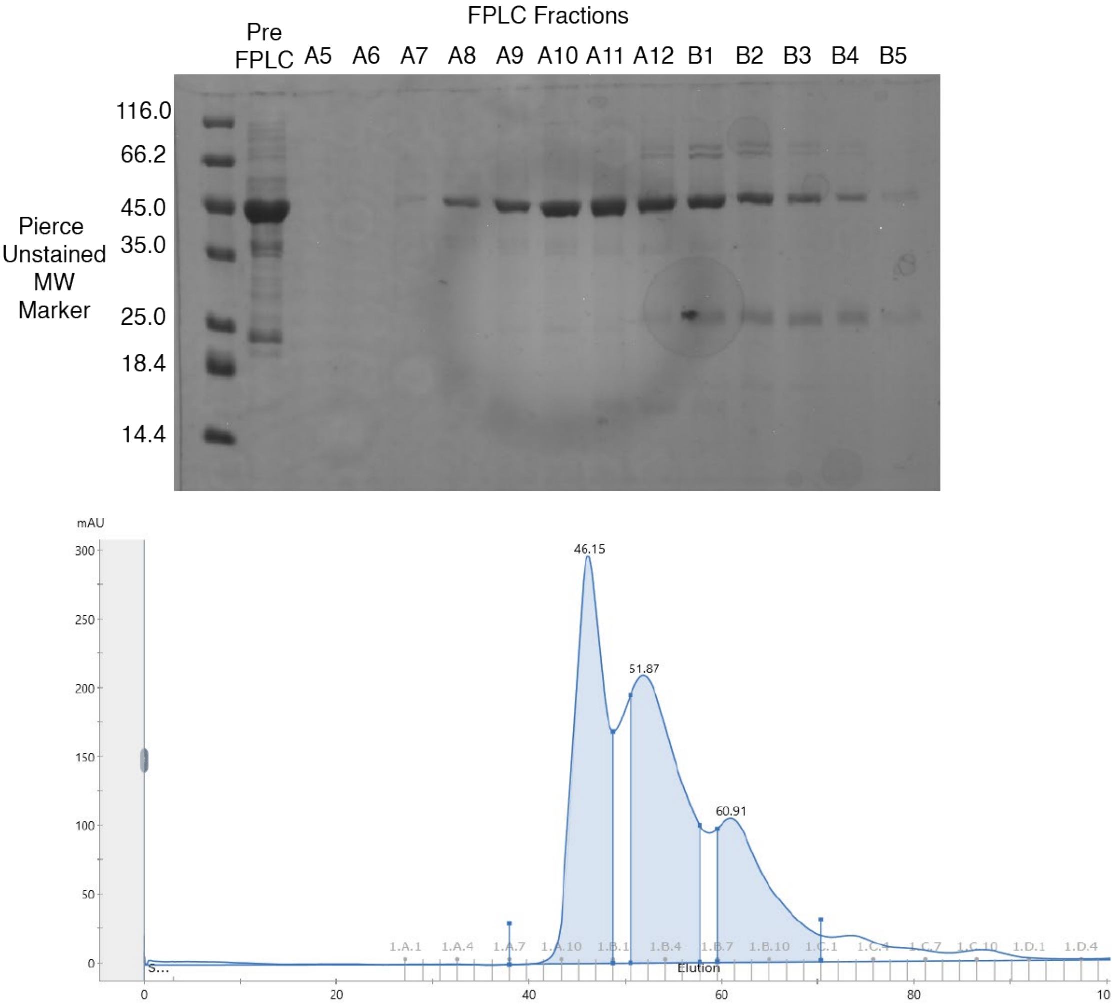

Figure S9 Purification of FabF. FabF was purified as described in the methods. The first peak on the FPLC was identified as the likely peak of interest. A sample of nickel purified FabF before the FPLC purification was run on the gel as a standard. A 12% polyacrylamide denaturing SDS gel was run in order to identify the useable fractions. Fractions A8-A12 were identified as sufficiently clean to concentrate for the experiment.

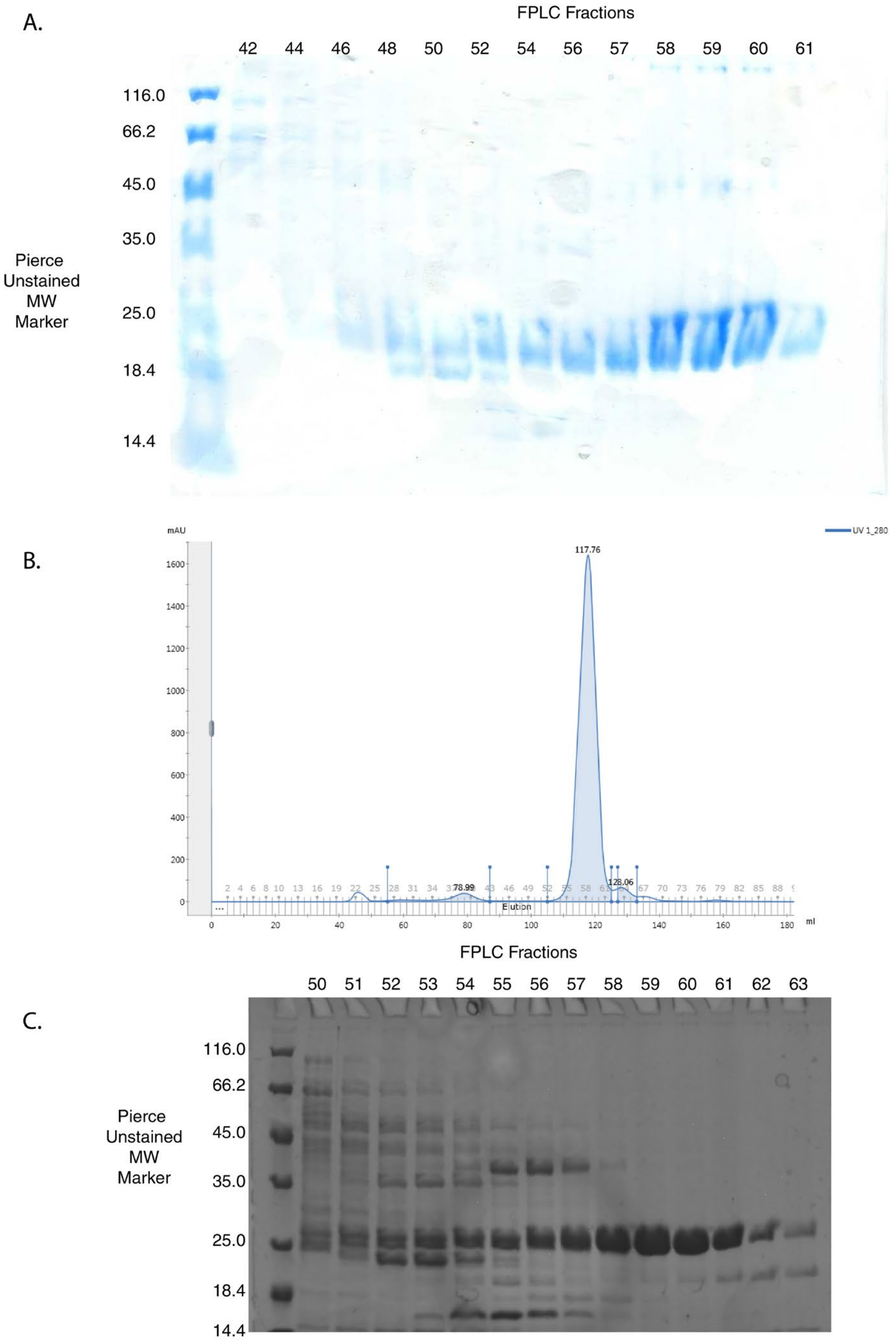

Figure S10 Purification of TesA. A/B) TesA was purified as described in the methods. The primary peak was sampled in order to ensure that the sample used was pure. A 12% polyacrylamide denaturing SDS gel was run focusing on the fractions making up the peak. Fractions 57, 58, 59, and 60 were collected and concentrated for the titration experiment. C) A test purification prior to the NMR experiment. This PAGE gel displayed an unsmeared example of the results expected for a TesA purification.

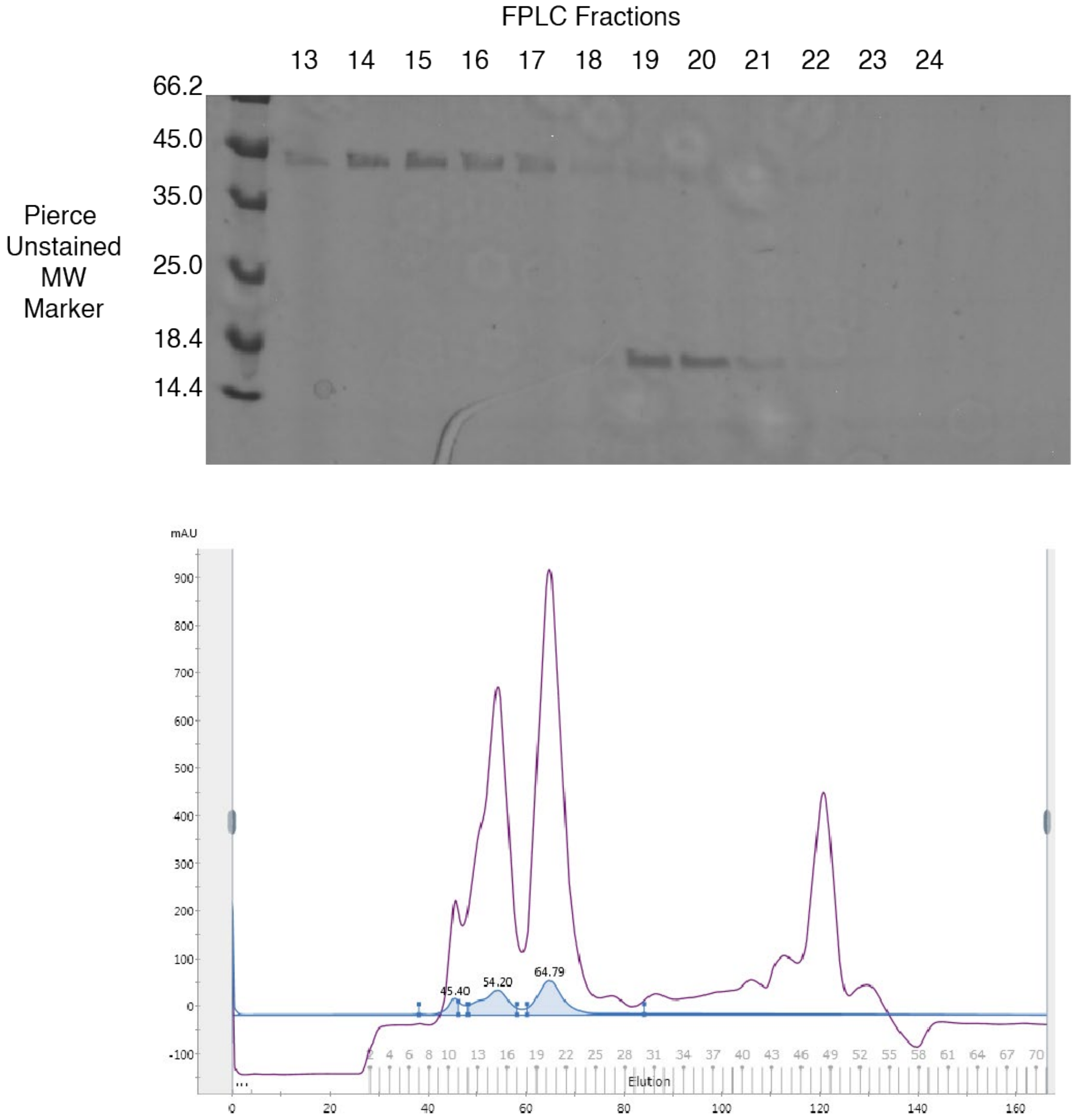

Figure S11 Purification of AcpP. An example of the purification of AcpP before an NMR experiment. Octanoyl-loaded AcpP was purified fresh for each titration. A 12% acrylamide denaturing SDS gel is shown. AcpP has very little absorbance at 280nm, as such the absorbance at 214 is also shown in magenta to help locate the AcpP's fractions. AcpP can be seen in fractions 19, 20, and 21, characteristically it travels unusually high on an SDS gel.

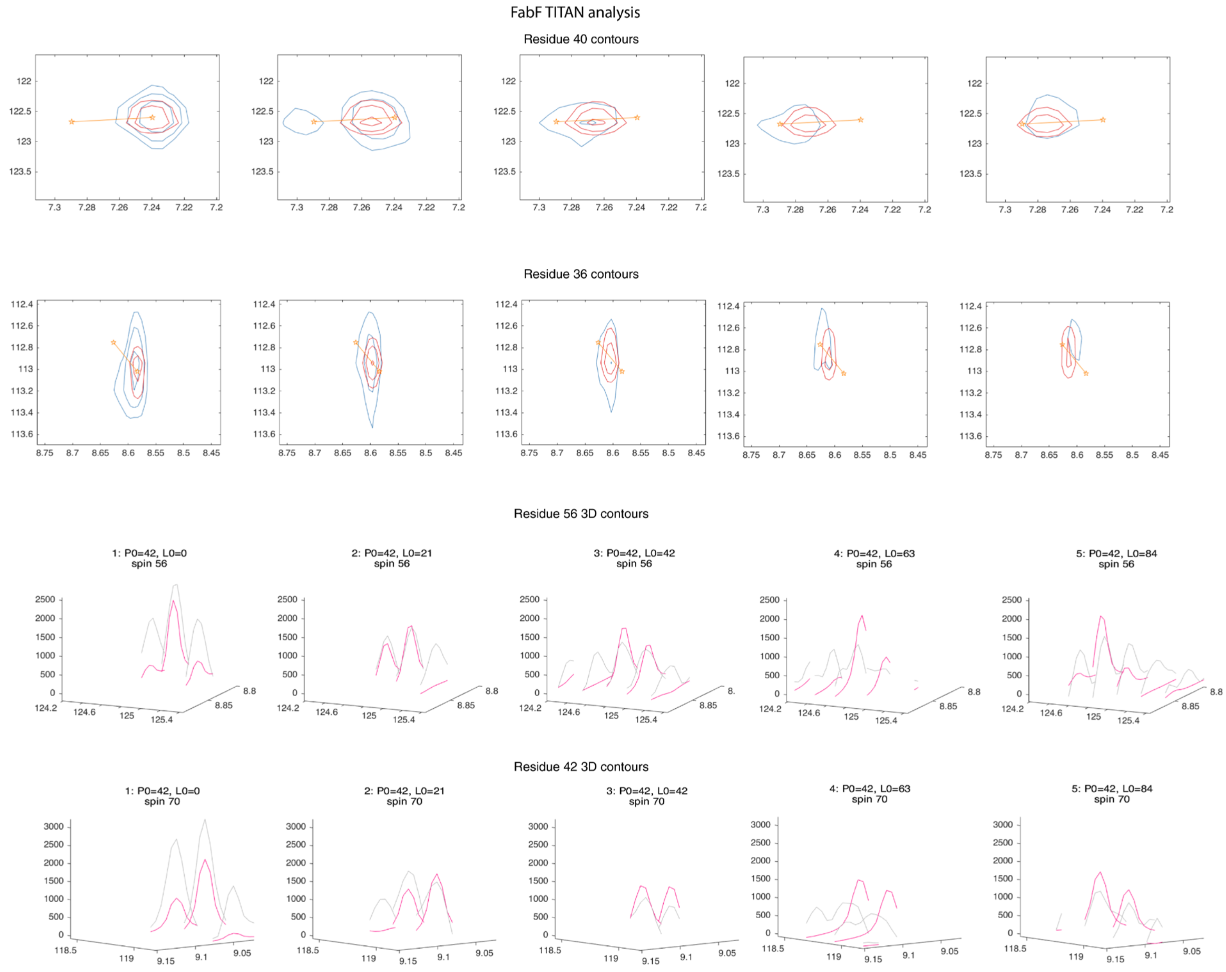

Figure S12 Titan peak fitting of FabF titration data. The FabF spectra and models were exported from the TITAN analysis program directly. Analysis was performed using the flexible docking method. Error analysis was performed using 300 steps of bootstrap error analysis. There was signal loss in the FabF experiment, likely due to the instability of the FabF protein and the presence of crashed partner protein in the titration.

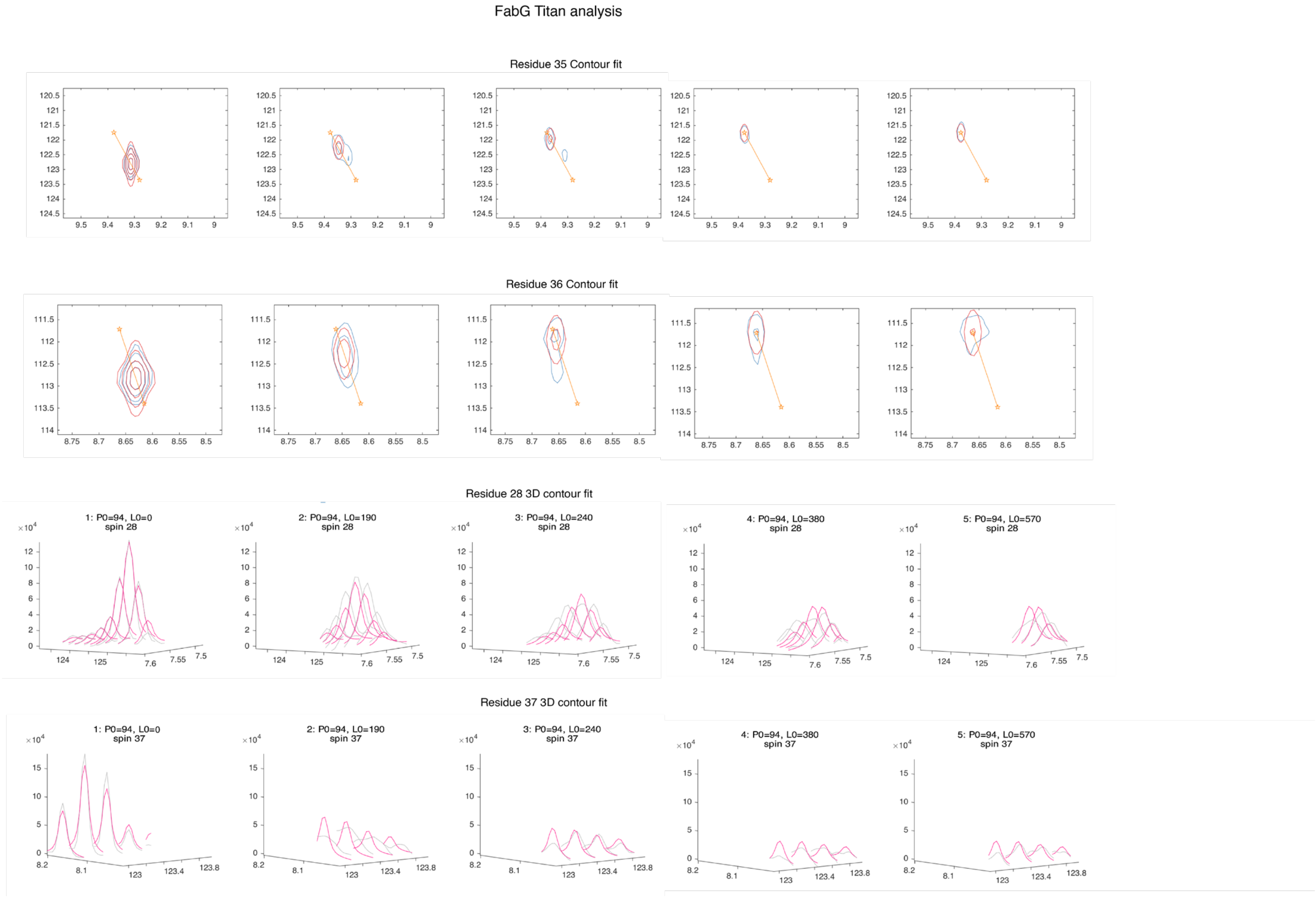

Figure S13 Titan peak fitting of FabG titration data. The FabG spectra and models were exported from the TITAN analysis program directly. Analysis was performed using the flexible docking method. Error analysis was performed using 300 steps of bootstrap error analysis.

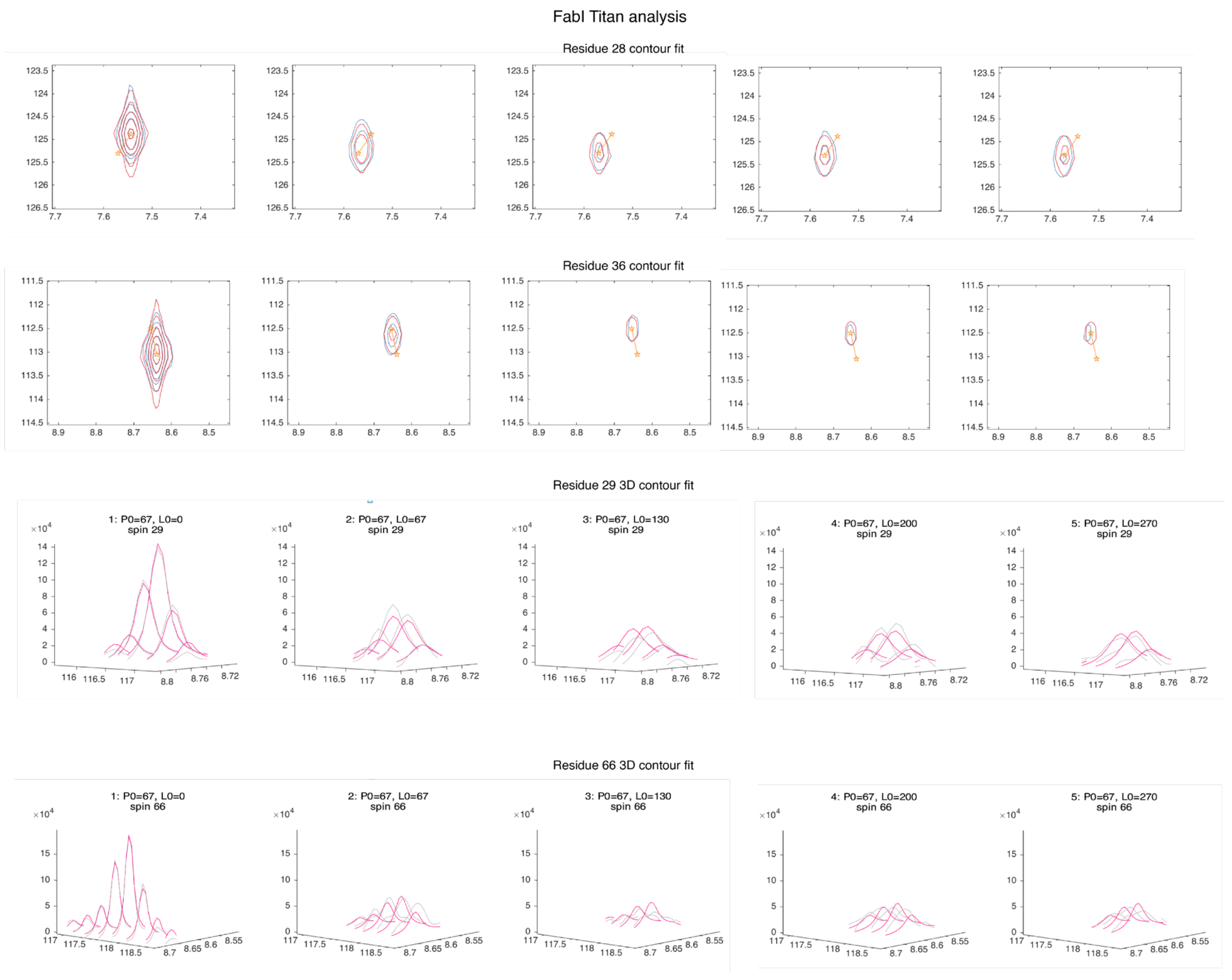

Figure S14 Titan peak fitting of FabI titration data. The FabI spectra and models were exported from the TITAN analysis program directly. Analysis was performed using the flexible docking method. Error analysis was performed using 300 steps of bootstrap error analysis

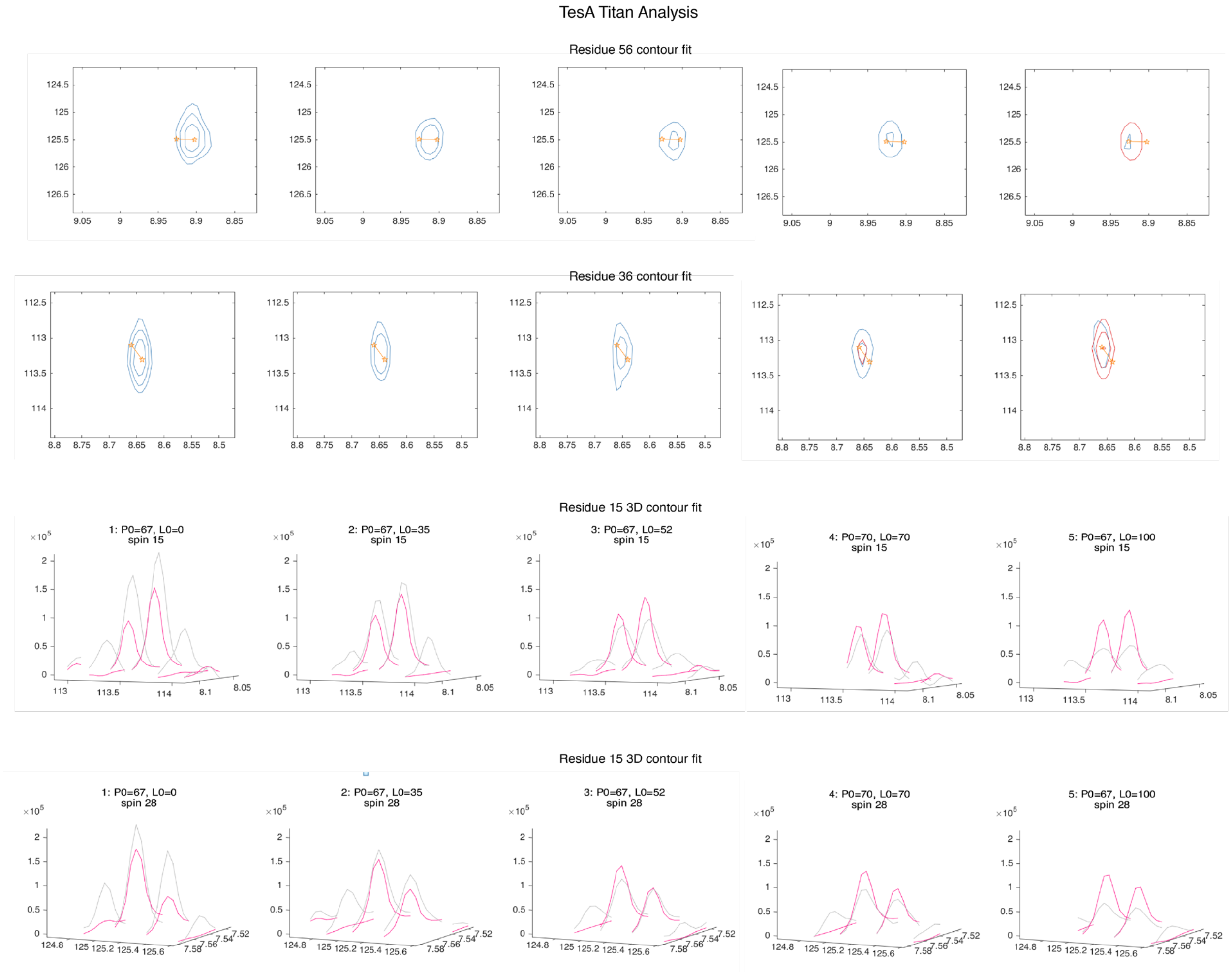

Figure S15 Titan peak fitting of TesA titration data. The TesA spectra and models were exported from the TITAN analysis program directly. Analysis was performed using the flexible docking method. Error analysis was performed using 300 steps of bootstrap error analysis

Table S1 Interface and global alignment scores for docked models. Alignments are generated from ICM docked models and aligned to crosslinked structures. The "informed" ACP residues are supplied form NMR titration data and the "informed" partner residues were taken from published data on essential electrostatic residues when possible.

Partner	Interface alignment (Å)	Global alignment (Å)	ACP "informed" residues supplied	Partner "informed" residues supplied
**FabA**	1.10	7.4	39,41,47	132,136
**FabZ**	1.19	5.39	N/A	N/A
**FabB**	0.84	8.5	35,38	61,62,65
**FabF**	1.58	6.7	35,39	65,212
**FabI**	N/A	N/A	35,44,47	201,204,205
**FabG**	N/A	N/A	35,39,47	14
**TesA**	N/A	N/A	31,44	N/A

Table S2 Table of the docked partner characteristics. Surface area is generated from the pymol “get area” command. Energetics values are taken directly from ICM.

Partner Enzyme	Surface area (Å^2)	Total binding energetics	Van der waals energetics	Electrostatic energetics
**FabF**	1022	-41.4	-42.8	-13
**FabB**	963	-49.32	-39.9	-12.7
**FabI**	1040	-46.4	-48.5	-11.83
**FabG**	1040	-53.8	-59.6	-8.5
**FabA**	704	-41.1	-28.5	-16.0
**FabZ**	718	-58.3	-41.1	-17.9
**TesA**	850	-41.85	-46	-6.9

Table S3: Peak list of zero point and saturated FabF titration points. CSPs were calculated form these chemical shifts using the CSP equation: $CSP = \sqrt{\frac{1}{2}[\delta_H^2 + (\alpha \cdot \delta_N^2)}$

RESIDUE	ZERO POINT ^1H PEAK	ZERO POINT ^{15}N PEAK	SATURATED ^1H PEAK	SATURATED ^{15}N PEAK
03I	8.57	121.215	8.574	121.214
04E	8.532	118.385	8.522	118.413
05E	7.782	117.169	7.753	117.067
06R	8.271	119.589	8.272	119.731
07V	8.847	118.887	8.869	118.731
08K	8.152	116.799	8.152	116.683
09K	8.199	120.264	8.188	120.226
10I	7.561	119.173	7.566	118.388
11I	8.243	118.641	8.226	118.693
12G	8.399	105.015	8.352	105.058
13E	8.142	119.956	8.109	120.23
14Q	8.346	117.156	8.441	117.299
15L	8.024	113.236	8.012	112.196
16G	7.745	109.597	7.716	109.732
17V	7.812	114.458	7.808	113.903
18K	8.466	122.713	8.483	122.49
19Q	8.704	122.536	8.705	122.367
20E	9.329	116.26	9.324	116.48
21E	7.81	116.694	7.787	116.658
22V	7.464	122.103	7.474	122.087
23T	7.27	115.423	7.229	115.118
24N	8.532	118.385	8.522	118.413
25N	8.026	111.683	8.024	111.458
26A	7.236	122.607	7.273	122.529
27S	9.871	116.772	9.9	117.013
28F	7.498	124.698	7.494	125.616
29V	8.682	116.397	8.638	116.655
30E	8.218	116.529	8.214	116.429
31D	7.709	113.747	7.766	113.265
32L	7.308	115.187	7.282	115.26
33G	7.158	106.181	7.303	106.436
34A	8.393	122.45	8.421	122.644
35D	9.237	122.792	9.256	121.955
36S	8.588	112.988	8.608	112.672
37L	8.114	123.253	8.13	123.138
38D	8.271	119.589	8.291	119.815
39T	8.116	111.653	8.055	112.295
40V	7.174	120.936	7.169	121.703
41E	7.725	119.111	7.784	119.1
42L	8.351	121.134	8.285	120.999
43V	7.925	118.949	7.934	118.975

**44M**	7.727	116.101	7.749	116.589
**45A**	8.059	120.675	8.016	120.219
**46L**	8.318	119.97	8.303	120.187
**47E**	8.581	119.522	8.506	119.389
**48E**	7.81	116.694	7.787	116.588
**49E**	7.871	119.406	7.884	119.412
**50F**	7.702	111.944	7.709	111.952
**51D**	7.809	122.137	7.777	121.763
**52T**	7.971	112.022	8.012	112.196
**53E**	8.049	122.387	8.037	122.645
**54I**	10.325	129.462	10.293	128.625
**55P**	0	0	0	0
**56D**	8.823	124.934	8.847	124.753
**57E**	9.172	115.993	9.26	116.394
**58E**	7.156	115.712	7.146	115.849
**59A**	8.114	123.253	8.13	123.138
**60E**	7.493	112.037	7.485	112.4
**61K**	6.969	113.744	7.023	113.929
**62I**	7.569	122.511	7.57	121.944
**63T**	7.971	112.022	8.012	112.196
**64T**	7.171	110.106	7.031	110.465
**65V**	7.931	121.163	7.911	120.502
**66Q**	8.616	117.698	8.458	117.439
**67A**	7.725	119.111	7.807	119.385
**68A**	7.89	122.432	7.82	121.446
**69I**	8.053	118.918	8.005	118.68
**70D**	9.051	118.888	9.088	118.642
**71Y**	8.105	121.417	8.101	120.881
**72I**	8.122	120.58	8.1	120.905
**73N**	8.769	117.956	8.77	117.97
**74G**	7.779	104.714	7.749	104.743
**75H**	7.587	118.351	7.566	118.388

Table S4: Peak list of zero point and saturated FabI titration points. CSPs were calculated form these chemical shifts using the CSP equation: $CSP = \sqrt{\frac{1}{2}[\delta_H^2 + (\alpha \cdot \delta_N^2)}$

RESIDUE	ZERO POINT ^{1}H PEAK	ZERO POINT ^{15}N PEAK	SATURATED ^{1}H PEAK	SATURATED ^{15}N PEAK
03I	8.627	121.2	8.619	121.18
04E	8.595	118.32	8.589	118.32
05E	7.838	117.15	7.833	117.1
06R	8.329	119.57	8.342	119.71
07V	8.903	118.83	8.897	118.88
08K	8.208	116.76	8.211	116.82
09K	8.261	120.12	8.24	120.24
10I	7.605	119.06	7.628	118.69
11I	8.293	118.63	8.246	118.64
12G	8.448	105	8.436	104.99
13E	8.188	119.89	8.18	119.9
14Q	8.4	117.11	8.378	117.13
15L	8.079	113.21	8.005	112.85
16G	7.793	109.61	7.788	109.59
17V	7.864	114.42	7.848	114.17
18K	8.526	122.69	8.523	122.65
19Q	8.765	122.47	8.759	122.37
20E	9.389	116.29	9.373	116.25
21E	7.864	116.7	7.851	116.66
22V	7.515	122.09	7.516	122.09
23T	7.328	115.36	7.33	115.3
24N	8.567	118.43	8.57	118.36
25N	8.085	111.71	8.076	112.03
26A	7.29	122.57	7.306	122.54
27S	9.929	116.71	9.947	116.83
28F	7.544	124.71	7.571	125.19
29V	8.746	116.41	8.754	116.63
30E	8.281	116.48	8.293	116.42
31D	7.767	113.67	7.787	113.48
32L	7.346	115.16	7.333	115.33
33G	7.225	106.21	7.259	106.26
34A	8.462	122.48	8.467	122.47
35D	9.294	122.74	9.279	122.32
36S	8.64	112.89	8.664	112.34
37L	8.157	123.31	8.056	123.44
38D	8.329	119.57	8.342	119.71
39T	8.158	111.54	8.13	111.27
40V	7.221	120.84	7.2	120.74
41E	7.796	118.85	7.838	118.99
42L	8.383	121.05	8.434	121.01
43V	7.952	118.77	7.944	119.31
44M	7.74	116.68	7.851	116.66
45A	8.11	120.75	8.177	120.36
46L	8.377	120.16	8.362	119.87

**47E**	8.669	119.5	8.558	119.26
**48E**	7.838	117.15	7.833	117.1
**49E**	7.944	119.12	7.944	119.31
**50F**	7.788	111.22	7.764	111.4
**51D**	7.849	122.06	7.838	121.96
**52T**	8.039	111.93	8.07	112.09
**53E**	8.097	122.31	8.076	122.4
**54I**	0	0	0	0
**55P**	0	0	0	0
**56D**	8.884	125.03	8.884	124.65
**57E**	9.24	115.94	9.292	116.15
**58E**	7.207	115.63	7.208	115.66
**59A**	8.157	123.31	8.251	123.66
**60E**	7.549	112.13	7.545	112.43
**61K**	7.007	113.71	7.048	113.91
**62I**	7.628	122.43	7.59	122.11
**63T**	8.039	111.93	8.07	112.09
**64T**	7.207	110.13	7.162	110.37
**65V**	7.988	121.12	7.998	120.92
**66Q**	8.667	117.66	8.549	117.51
**67A**	7.796	118.85	7.853	118.99
**68A**	7.946	122.39	7.947	122.86
**69I**	8.096	118.94	8.056	118.67
**70D**	9.107	118.78	9.131	118.64
**71Y**	8.123	121.12	8.125	121.13
**72I**	8.188	119.89	8.18	119.9
**73N**	8.81	117.79	8.809	117.85
**74G**	7.832	104.75	7.811	104.97
**75H**	7.644	118.42	7.628	118.69

Table S5: Peak list of zeropoint and saturated FabG titration points. CSPs were calculated form these chemical shifts using the CSP equation: $CSP = \sqrt{\frac{1}{2}[\delta_H^2 + (\alpha \cdot \delta_N^2)}$

RESIDUE	ZERO POINT ^{1}H PEAK	ZERO POINT ^{15}N PEAK	SATURATED ^{1}H PEAK	SATURATED ^{15}N PEAK
03I	8.616	121.22	8.623	121.22
04E	8.585	118.32	8.595	118.34
05E	7.836	117.12	7.841	117.13
06R	8.329	119.61	8.341	119.69
07V	8.893	118.76	8.899	118.86
08K	8.209	116.75	8.243	116.87
09K	8.244	120.33	8.232	120.23
10I	7.605	119.12	7.636	118.75
11I	8.291	118.63	8.236	118.89
12G	8.451	105	8.447	105.12
13E	8.188	119.93	8.197	119.92
14Q	8.396	117.13	8.382	117.2
15L	8.075	113.17	7.992	112.96
16G	7.794	109.6	7.789	109.64
17V	7.862	114.35	7.854	114.18
18K	8.525	122.66	8.536	122.63
19Q	8.766	122.45	8.775	122.37
20E	9.377	116.25	9.388	116.32
21E	7.859	116.68	7.861	116.66
22V	7.516	122.11	7.523	122.09
23T	7.31	115.27	7.334	115.31
24N	8.585	118.32	8.595	118.34
25N	8.082	111.7	8.065	110.94
26A	7.29	122.55	7.315	122.56
27S	9.929	116.69	9.938	116.93
28F	7.538	124.68	7.533	125.43
29V	8.747	116.37	8.735	116.54
30E	8.275	116.47	8.295	116.43
31D	7.76	113.68	7.793	113.38
32L	7.353	115.12	7.375	115.44
33G	7.207	106.2	7.304	106.25
34A	8.449	122.38	8.449	122.5
35D	9.315	122.83	9.379	121.71
36S	8.631	112.84	8.657	111.64
37L	8.163	123.24	8.048	123.44
38D	8.311	119.38	8.341	119.69
39T	8.166	111.54	8.154	112.13
40V	7.21	120.78	7.243	121.79
41E	7.797	118.79	7.848	119.18
42L	8.388	121.09	8.445	120.88
43V	7.975	118.92	7.958	119.28
44M	7.729	116.63	7.771	116.38
45A	8.122	121.22	8.127	121.11

**46L**	8.365	120.21	8.374	120.13
**47E**	8.677	119.63	8.558	119.97
**48E**	7.834	116.81	7.841	117.13
**49E**	7.93	119.38	7.958	119.28
**50F**	7.782	111.24	7.731	111.84
**51D**	7.843	121.98	7.789	121.74
**52T**	8.027	111.95	8.084	111.75
**53E**	8.081	122.23	8.072	122.36
**54I**	0	0	0	0
**55P**	0	0	0	0
**56D**	8.883	124.98	8.953	124.78
**57E**	9.237	115.95	9.285	116.15
**58E**	7.201	115.67	7.189	115.68
**59A**	8.15	122.45	8.251	122.66
**60E**	7.545	111.96	7.548	112.42
**61K**	7.011	113.85	7.003	113.45
**62I**	7.612	122.39	7.606	121.99
**63T**	8.027	111.95	8.084	111.75
**64T**	7.215	110.14	7.086	110.4
**65V**	7.98	121.12	8.029	120.92
**66Q**	8.666	117.62	8.595	118.34
**67A**	7.764	118.95	7.818	119.13
**68A**	7.931	122.34	7.866	121.43
**69I**	8.094	118.79	8.069	118.69
**70D**	9.098	118.77	9.152	118.58
**71Y**	8.122	121.22	8.127	121.11
**72I**	8.188	120.44	8.179	120.58
**73N**	8.806	117.94	8.812	117.92
**74G**	7.817	105.03	7.812	105.04
**75H**	7.637	118.7	7.636	118.75

Table S6: Peak list of zero point and saturated TesA titration points. CSPs were calculated form these chemical shifts using the CSP equation: $CSP = \sqrt{\frac{1}{2}[\delta_H^2 + (\alpha \cdot \delta_N^2)}$

RESIDUE	ZERO POINT ^{1}H PEAK	ZERO POINT ^{15}N PEAK	SATURATED ^{1}H PEAK	SATURATED ^{15}N PEAK
03I	8.641	121.46	8.638	121.45
04E	8.603	118.64	8.601	118.64
05E	7.837	117.36	7.836	117.34
06R	8.332	119.72	8.336	119.86
07V	8.907	119.03	8.903	118.95
08K	8.219	117.09	8.222	117.11
09K	8.269	120.41	8.26	120.39
10I	7.613	119.29	7.616	119.31
11I	8.308	118.87	8.299	118.89
12G	8.472	105.3	8.47	105.32
13E	8.198	120.09	8.197	120.04
14Q	8.401	117.38	8.388	117.25
15L	8.085	113.38	8.072	113.35
16G	7.807	109.75	7.802	109.73
17V	7.87	114.55	7.858	114.44
18K	8.528	123.03	8.527	122.99
19Q	8.781	122.87	8.783	122.82
20E	9.409	116.51	9.416	116.53
21E	7.87	116.91	7.864	116.89
22V	7.521	122.25	7.518	122.22
23T	7.325	115.54	7.325	115.46
24N	8.576	118.78	8.601	118.64
25N	8.085	111.91	8.073	111.96
26A	7.288	122.8	7.289	122.76
27S	9.929	116.91	9.931	117.15
28F	7.553	124.99	7.557	125.03
29V	8.753	116.61	8.757	116.64
30E	8.297	116.73	8.298	116.71
31D	7.793	114.38	7.759	113.74
32L	7.349	115.35	7.325	115.46
33G	7.223	106.4	7.225	106.38
34A	8.447	122.59	8.448	122.55
35D	9.304	123.07	9.308	122.91
36S	8.646	113.13	8.654	112.93
37L	8.167	123.47	8.16	123.45
38D	8.332	119.72	8.336	119.86
39T	8.162	111.74	8.156	111.58
40V	7.222	120.99	7.224	120.97
41E	7.805	119.03	7.829	119.16
42L	8.396	121.29	8.399	120.99
43V	7.974	119.03	7.986	119.14
44M	7.758	116.92	7.864	116.89
45A	8.128	121.39	8.129	120.94
46L	8.383	120.4	8.389	120.38

**47E**	8.694	119.86	8.678	119.74
**48E**	7.843	116.94	7.864	116.89
**49E**	7.939	119.55	7.949	119.4
**50F**	7.779	111.41	7.767	111.52
**51D**	7.862	122.21	7.851	122.13
**52T**	8.018	112.08	8.063	112.12
**53E**	8.109	122.44	8.108	122.4
**54I**	0	0	0	0
**55P**	0	0	0	0
**56D**	8.906	125.3	8.923	125.33
**57E**	9.253	116.19	9.287	116.14
**58E**	7.21	115.89	7.201	115.91
**59A**	8.175	122.84	8.177	122.84
**60E**	7.549	112.19	7.551	112.3
**61K**	7.024	113.99	7.022	113.94
**62I**	7.62	122.63	7.62	122.46
**63T**	8.018	112.08	7.982	112.24
**64T**	7.231	110.21	7.217	110.22
**65V**	7.988	121.4	7.988	121.33
**66Q**	8.676	117.92	8.667	117.88
**67A**	7.775	119.23	7.782	119.2
**68A**	7.938	122.63	7.932	122.54
**69I**	8.111	119.03	8.103	118.98
**70D**	9.107	119.08	9.109	119.06
**71Y**	8.156	121.59	8.151	121.45
**72I**	8.192	120.69	8.195	120.64
**73N**	8.832	118.2	8.825	118.18
**74G**	7.826	104.93	7.828	104.96
**75H**	7.64	118.56	7.643	118.6

Table S7: Comparisons of the residue informed and uninformed top 10 docking poses. The 10 most energetically favorable poses from otherwise identical “informed” and “uninformed” docking calculations.

Pose	FabF "informed" (Å)	FabF "uninformed" (Å)	FabB "informed" (Å)	FabB "uninformed" (Å)	FabA "informed" (Å)	FabA "uninformed" (Å)
1	6.7	14.8	8.5	8.5	7.4	7.4
2	9.2	17.7	2.9	19.9	12.9	12.9
3	5.8	26.2	6.7	4.5	7.8	7.8
4	7.4	26	4.2	11.7	6.7	6.7
5	10.4	10.4	4.1	12.2	6.8	6.8
6	8.5	21.7	4.5	17.5	12.8	12.8
7	8.1	19.1	6.2	7.8	8	13.2
8	4.2	22.3	6.3	6.8	13.2	5.8
9	9.3	22.4	4.7	8.5	5.8	18.4
10	10.2	20.2	7.6	7.9	18.4	7.1
Average	7.98	20.08	5.57	10.53	9.98	9.89

Table S8: Comparisons of online docking resources' ability to recreate the FabF-AcpP crosslinked interface. RMSD calculations were performed using the same methods as the ICM docked structures. All calculations were performed as described in the methods on free to use docking websites.

Software scoring rank	ClusPro balanced scoring RMSD (Å)	ClusPro electrostatic scoring RMSD (Å)	Rosie: refining Cluspro electrostatic rank 1 structure RMSD (Å)	HADDOCK ICM prepared files RMSD (Å)	HADDOCK raw PDB files RMSD (Å)
1	12.98	4.56	4.54	6.4	17.4
2	14.95	10.06	4.53	6	17.7
3	12.77	7.7	4.57	6.33	17.6

Table S9: Thermodynamic and Kinetic parameters of the elongating enzymes and a thioesterase of *E. coli* Fatty Acid Biosynthesis binding an amide linked octanoyl AcpP. Parameters were calculated using the TITAN lineshape analysis software.

Protein	K_d (μM)	K_{off} (s^{-1})	n
**FabF**	6.9±8.5	3350±3310	1.3±0.3
**FabB**	37.6±6.6		
**FabI**	1.6±1.2	6360±3260	1.2±0.1
**FabG**	52.3±27.5	3559±2061	2.2±0.4
**TesA**	13±8	9750±850	monomer
