# Supplementary material for: Elucidation of transient protein-protein interactions within carrier protein-dependent biosynthesis
Source: Commun Biol. 2021 Mar 16;4:340. doi: 10.1038/s42003-021-01838-3 (PMC7966745; doi:10.1038/s42003-021-01838-3)
Supplement: Supplementary file 3 — Description of Additional Supplementary Files [file 42003_2021_1838_MOESM3_ESM.pdf]

Description of Additional Supplementary Files

File name: Supplementary Data 1-4

File Description:

Supplementary data 1: Peak list of zero point and saturated FabF titration points. CSPs were calculated form these chemical shifts using the CSP equation:

$$CSP = \sqrt{\frac{1}{2}[\delta_H^2 + (\alpha \cdot \delta_N^2)}$$

Supplementary data 2: Peak list of zero point and saturated FabI titration points. CSPs were calculated form these chemical shifts using the CSP equation:

$$CSP = \sqrt{\frac{1}{2}[\delta_H^2 + (\alpha \cdot \delta_N^2)}$$

Supplementary data 3: Peak list of zeropoint and saturated FabG titration points. CSPs were calculated form these chemical shifts using the CSP equation:

$$CSP = \sqrt{\frac{1}{2}[\delta_H^2 + (\alpha \cdot \delta_N^2)}$$

Supplementary data 4: Peak list of zero point and saturated TesA titration points. CSPs were calculated form these chemical shifts using the CSP equation:

$$CSP = \sqrt{\frac{1}{2}[\delta_H^2 + (\alpha \cdot \delta_N^2)}$$

File name: Supplementary Data 5

File Description:

Protein-protein docked models-

For all of the files the multiple partner protein chains were merged to chain A, as the program docks only single chains. Ranking provided is based on the overall energetics “energy” scoring performed by the ICM FFT protein-protein docking.
