# Supplementary material for: Elucidation of transient protein-protein interactions within carrier protein-dependent biosynthesis
Source: Commun Biol. 2021 Mar 16;4:340. doi: 10.1038/s42003-021-01838-3 (PMC7966745; doi:10.1038/s42003-021-01838-3)
Supplement: Supplementary file 5 — Supplementary Data 5 [file 42003_2021_1838_MOESM5_ESM.zip › Protein_protein_docked_models/Protein_protein_docked_models/LEGEND_readme.docx]

**Title:** Elucidation of transient protein-protein interactions within carrier protein-dependent biosynthesis

**Authors:** Thomas G. Bartholow^1^, Terra Sztain^1^, Ashay Patel^1^, D. John Lee^1,3^, Megan A. Young^1^, Ruben Abagyan^2^, Michael D. Burkart^1*^

For all of the files the multiple partner protein chains were merged to chain A, as the program docks only single chains. Ranking provided is based on the overall energetics “energy” scoring performed by the ICM FFT protein-protein docking.

https://www.molsoft.com/gui/protprot.html
